# Supplementary material for: Regulation of KIR3DL3 Expression via miRNA
Source: Genes (Basel). 2019 Aug 9;10(8):603. doi: 10.3390/genes10080603 (PMC6723774; doi:10.3390/genes10080603)
Supplement: Supplementary file 1 [file genes-10-00603-s001.pdf]

Supplementary materials and methods

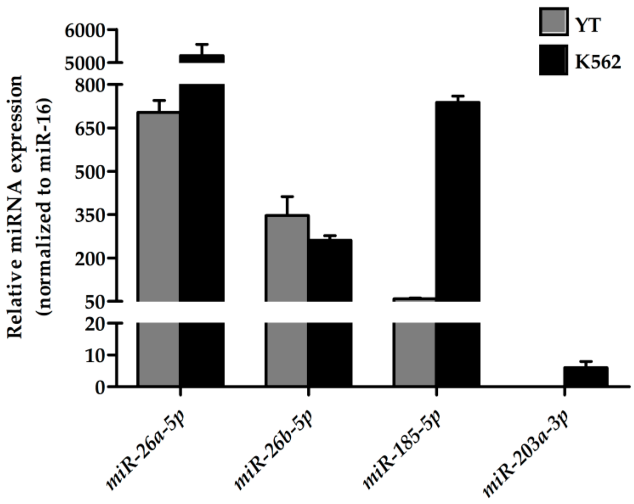

Figure S1. Endogenous miRNA expression levels in the YT and K562 cell lines. Relative candidate miRNA expressions in YT and K562 are presented.The expression of miR-203a-3p was not detected in YT but very small amount was present in K562.

Table S1. Primer sets for KIR3DL3 and miRNA detection.

Primer name	Primer Sequences 5′-3′
**Specific primers for *qRT-PCR* of the genes investigated**	
2DL4	F: TCAGGACAAGCCCTTCTGC
	R: GACAGGGACCCCATCTTTC
3DL1	F: TGAGCACTTCTTTCTGCACAA
	R: TAGGTCCCTGCAAGGGCAA
3DL3	F: GCAATGTTGGTCAGATGTCAG
	R: AGCCGACAACTCATAGGGTA
RPII	F: AGTATGGCATGGAGATCCCC
	R: ATAGGCAGGGGTTGCACC
**Primer set for 3′UTR KIR3DL3 mutagenesis**	
3′UTR WT	F: CACCGGATCCCACGGAACTTCCAAATGCTGAGCG
	R: CACCCTCGAGATGAGGGCAGACATGTTTAC
Mu_miR-26a/b-5p (295)	F: TCCATTTCACCTAACCCCTGCC
	R: GGCAGGGGTTAGGTGAAATGGA
Mu_miR-26a/b-5p (344)	F: TCCTAGTCTGCTTAAGGCTGCGAT
	R: ATCGCAGCCTTAAGCAGACTAGGA
Mu_ 185-5p	F: CTGCCCACCTATCGAACCTAACT
	R: AGTTAGGTTCGATAGGTGGGCAG
Mu_203a-3p	F: CAATTCTCCAGTACACTTGACCCC
	R: GGGGTCAAGTGTACTGGAGAATTG
**Stem-loop primer set for miRNA detection**	
S-hsa-miR-26a-5p	S: GTCGTATCCAGTGCAGGGTCCGAGGTATTCGCACTGGATACGACAGCCTA
S-hsa-miR-26b-5p	S: GTCGTATCCAGTGCAGGGTCCGAGGTATTCGCACTGGATACGACACCTAT
S-hsa-miR-185-5p	S: GTCGTATCCAGTGCAGGGTCCGAGGTATTCGCACTGGATACGACTCAGGA
S-hsa-miR-203a-3p	S: GTCGTATCCAGTGCAGGGTCCGAGGTATTCGCACTGGATACGACCTAGTG
F-hsa-miR-26a-5p	F: GCGTGCTTCAAGTAATCCAGGA
F-hsa-miR-26b-5p	F: CCGCGCTTCAAGTAATTCAGG
F-hsa-miR-185-5p	F: CCGCTGGAGAGAAAGGCAGT
F-hsa-miR-203a-3p	F: CCGCCGTGAAATGTTTAGGAC
Universal Reverse	R: GTGCAGGGTCCGAGGT

Table S2. Killer Immunoglobulin-like receptor (KIR) expression profiles in the NK-92 cell line.

	**Killer-cell immunoglobulin-like receptor (KIR)**													
	**2DL 1**	**2DL 2**	**2DL 3**	**2DL 4**	**2DL 5**	**2DS 1**	**2DS 2**	**2DS 3**	**2DS 4**	**2DS 5**	**3DL 1**	**3DL 2**	**3DL 3**	**3DS 1**
DNA	+	+	+	+	-	-	+	-	F/D	-	+	+	+	-
mRN A	-	-	-	+	-	-	-	-	-	-	+	-	+	-
Protei n	-	-	-	+	-	-	-	-	-	-	+	-	-	-

+: positive, -: negative, F: full form, D: deleted form (22 base pair deletion in KIR2DS4 exon 5)

Table S3. The percent sequence identity between the 3′ untranslated region (3′UTR) of different KIRs*.

	2D	2D	2D	2D	2D	2D	2D	2D	2D	2D	3D	3D	3D
2DL2*0010101	99												
2DL3*0010101	96	96											
2DL4*0010201	90	90	93										
2DL5A*0010101	90	90	92	93									
2DS1*0020101	91	91	92	87	88								
2DS2*0010101	91	91	92	87	87	99							
2DS3*00103	69	70	70	59	59	98	98						
2DS4*0010101	91	91	92	87	88	99	100	98					
2DS5*0020101	94	94	96	91	93	97	97	99	97				
3DL1*0010101	92	93	93	90	90	89	88	68	89	92			
3DL2*0010101	90	90	92	92	91	87	87	63	87	92	86		
3DL3*00101	88	89	90	86	86	94	94	79	94	95	88	86	
3DS1*0130101	97	98	96	90	90	96	96	96	96	95	92	90	93

All allelic 3′-UTR sequences of the KIR genes used for analysis were retrieved from a repository of the IPD-KIR Database (https://www.ebi.ac.uk/ipd/kir).

Table S4. Computational prediction of three candidate miRNAs binding on 3′UTR of 14 KIR genes.

KIRs	mfe (kcal/mol)[a]		
	**miR-26a-5p**	**miR-26b-5p**	**miR-185-5p**
**2DL1*0020101**	18.7/-24.0	-16.1/-20.9	-25.2
**2DL2*0010101**	-19.0/-24.0	-16.4/-20.9	-25.2
**2DL3*0010101**	-18.7/-24.0	-16.1/-20.9	-19.8/-25.2
**2DL4*0010201**	-18.7/-24.0	-16.1/-20.9	-22.5/-25.2
**2DL5A*0010101**	-18.7/-24.0	-16.1/-20.9	-25.2
**2DS1*0020101**	-18.7	-16.1	-25.2
**2DS2*0010101**	-18.7	-16.1	-25.2
**2DS3*0010301**	-18.7/-24.0	-16.1/-20.9	-25.2
**2DS4*0010101**	-18.7	-16.1	-25.2
**2DS5*0020101**	-18.7/-24.0	-16.1/-20.9	-25.2
**3DL1*0010101**	-19.0/-24.0	-16.4/-20.9	-25.2
**3DL2*0010101**	-17.9/-23.9	-15.0/-21.0	-25.2
**3DL3*00101**	-18.7//24.0	-16.1/-20.9	21.4/-25.2
**3DS1*0130101**	-19.0/-24.0	-16.4/-20.9	-25.2

[a] minimum free energy (mfe) of miRNA:mRNA duplex was calculated using RNAhybrid.

Table S5. Target genes for miR-26a-5p, -26b-5p and -185-5p.

miRNA	Target gene	Reference sequence	Gene name	Pathway
miR-26a/b-5p	JUN	NM_002228	Jun oncogene	Proapoptotic
	BAX	NM_004324	BCL2-associated X protein	Proapoptotic
	BIRC2	NM_001166	Baculoviral IAP repeat-containing 2	Apoptosis
	BIRC3	NM_182962	Baculoviral IAP repeat-containing 3	Apoptosis
	XIAP	NM_001167	X-linked inhibitor of apoptosis	Apoptosis
	MCL1	NM_021960	Myeloid cell leukemia sequence 1	Antiapoptotic
	BID	NM_001196	BH3 interacting domain death agonist	Proapoptotic
	CYCS	NM_018947	Cytochrome c, somatic	Proapoptotic
	APAF1	NM_001160	Apoptotic peptidase activating factor 1	Proapoptotic
	TNFRSF10A	NM_003844	Tumor necrosis factor receptor superfamily, member 10a	Apoptosis
	TNFRSF11B	NM_002546	Tumor necrosis factor receptor superfamily, member 11b	Apoptosis
	TNFSF10	NM_003810	TNFSF10 tumor necrosis factor (ligand) superfamily, member 10	Apoptosis
miR-185-5p	Akt1	NM_005163	v-akt murine thymoma viral oncogene homolog 1	Cell survival
	IKBKB	NM_001556	Inhibitor of kappa light polypeptide gene enhancer in B-cells, kinase beta	Cell survival
	IKBKE	NM_014002	Inhibitor of kappa light polypeptide gene enhancer in B-cells, kinase epsilon	Cell survival
	IKBKG	NM_003639	Inhibitor of kappa light polypeptide gene enhancer in B-cells, kinase gamma	Cell survival
	PPP2CA	NM_002715	Protein phosphatase 2, catalytic subunit, alpha isozyme	Apoptosis
	PPP2CB	NM_004156	Protein phosphatase 2, catalytic subunit, beta isozyme	Apoptosis
	CAPN11	NM_007058	Calpain 11	Apoptosis
	CAPN7	NM_014296	Calpain 7	Apoptosis
	CAPNS1	NM_001749	Calpain, small subunit 1	Apoptosis
	MAP2K4	NM_003010	Mitogen-activated protein kinase kinase 4	Cell survival
	MAPK3	NM_002746	Mitogen-activated protein kinase 3	Cell survival
	E2F1	NM_005225	E2F transcription factor 1	Proapoptotic
	RHBDD1	NM_032276	Rhomboid domain containing 1	Antiapoptotic
	CIAPIN1	NM_020313	Cytokine induced apoptosis inhibitor 1	Apoptosis
	TNF	NM_000594	Tumor necrosis factor	Apoptosis
	NAIP	AK124511	NLR family, apoptosis inhibitory protein	Apoptosis
	BCL2L1	NM_001191	BCL2-like 1	Proapoptotic/ Antiapoptotic
	BIK	NM_001197	BCL2-interacting killer (apoptosis-inducing)	Proapoptotic
	BNIP3	NM_004052	BCL2/adenovirus E1B 19kDa interacting protein 3	Proapoptotic
	BCL2L14	NM_138722	BCL2-like 14 (apoptosis facilitator)	Proapoptotic
	BBC3	NM_001127240	BCL2 binding component 3	Antiapoptotic
	TNFRSF10B	NM_003842	Tumor necrosis factor receptor superfamily, member 10b	Apoptotic
	FAS	NM_000043	Fas cell surface death receptor	Apoptotic
	TNFRSF10C	NM_003841	Tumor necrosis factor receptor superfamily, member 10c, decoy without an intracellular domain	Apoptotic
	FADD	NM_003824	Fas (TNFRSF6)-associated via death domain	Apoptotic
miR-26a/b-5p+185-5p	BOD1	NM_138369	Biorientation of chromosomes in cell division 1	Proapoptotic
	BCL2	NM_000633	B-cell CLL/lymphoma 2	Proapoptotic/ Antiapoptotic
	PIM1	NM_001001852	Pim-1 oncogene	Cell survival
	PIM2	NM_006875	Pim-2 oncogene	Cell survival
	PIM 3	NM_001001852	Pim-3 oncogene	Cell survival
	TNFRSF10D	NM_003840	Tumor necrosis factor receptor superfamily, member 10d, decoy with truncated death domain	Apoptotic

miR-26a/b-5p+185-5p	PAK1	NM_002576	P21 protein (Cdc42/Rac)-activated kinase 1	Cell survival
	PAK1IP1	NM_017906	PAK1 interacting protein 1	Cell survival
	PAK2	NM_002577	P21 protein (Cdc42/Rac)-activated kinase 2	Cell survival
	PAK3	NM_002578	P21 protein (Cdc42/Rac)-activated kinase 3	Cell survival
	PAK4	NM_005884	P21 protein (Cdc42/Rac)-activated kinase 4	Cell survival
	PAK6	NM_020168	P21 protein (Cdc42/Rac)-activated kinase 6	Cell survival
	PAK7	NM_020341	P21 protein (Cdc42/Rac)-activated kinase 7	Cell survival
	BTRC	NM_003939	Beta-transducin repeat containing	Cell cycle
	FOXO3	NM_001455	Forkhead box O3	Apoptosis
	TP53	NM_000546	Tumor protein p53	Apoptosis
	TP63	NM_003722	Tumor protein p63	Apoptosis
	UCHL1	NM_004181	Ubiquitin carboxyl-terminal esterase L1 (ubiquitin thiolesterase)	Apoptosis
	HIF1A	NM_001530	Hypoxia-inducible factor 1, alpha subunit (basic helix-loop-helix transcription factor)	Proapoptotic
	HIF1AN	NM_017902	Hypoxia-inducible factor 1, alpha subunit inhibitor	Proapoptotic
	SMAD1	NM_005900	SMAD family member 1	Proapoptotic
